# VTE Prevention Ability Among Community Nurses in the Medical Consortium Based on the Kirkpatrick Mode Evaluation of the Effects of Training

**DOI:** 10.1111/phn.13479

**Published:** 2024-11-20

**Authors:** Weihong Fan, Bei Tian, Pei Xu, Zuoli Zou, Xiaoling Zhou, Yan Wu, Lianbao Wu, Yingbiao Wu, Peifeng Tang, Weiqun Liu

**Affiliations:** ^1^ Zhoupu Hospital Shanghai University of Medicine and Health Sciences Shanghai China; ^2^ School of Public Health The Key Laboratory of Environmental Pollution Monitoring and Disease Control Ministry of Education Guizhou Medical University Guiyang China

**Keywords:** community nurses, Kirkpatrick model, medical consortium, VTE prevention

## Abstract

**Purpose:**

To explore the application of the Kirkpatrick model in VTE prevention training among community nurses in the medical consortium.

**Method:**

A team of experts was established to formulate a VTE prevention ability training program for community nurses in the medical consortium. According to a literature review, the results of a questionnaire survey and the results of on‐site supervision surveys of nurses in five community health service centers in the medical consortium. The Kirkpatrick model was applied to train 117 nurses in five community service centers, and the effects on the four dimensions (i.e., the reaction layer, learning layer, behavior layer, and results layer) were observed.

**Results:**

A total of 100% of nurses’ reported satisfaction with the training, and the attendance rate was ≥98%. After the training, the theoretical knowledge of VTE prevention and the results of the skills assessment of community nurses in the medical consortium were significantly greater than the pretraining levels (*p* < 0.001). Three months after training, the nurses’ VTE prevention execution score significantly improved compared with the pretraining scores (*p* < 0.001). The compliance rate and standard rate of ankle pump movement significantly improved compared with those before training (*p* < 0.001).

**Conclusion:**

Training based on the offset model can effectively improve VTE prevention knowledge among community nurses in the medical consortium, thereby enhancing VTE prevention among patients and at improving the compliance and standardization of patients' self‐prevention. These findings provide a reference for future in‐service training programs targeting VTE prevention among nurses in community hospitals.

## 1 Introduction

The prevention of venous thromboembolism (VTE) has been widely studied, and for 4 consecutive years, China has listed VTE prevention as one of the top 10 goals for improving national medical quality and safety. Previous studies have shown that the annual incidence of VTE ranges from 0.75‰ to 2.69‰, and its trend in increasing annually. The high incidence and high mortality rate of VTE have become major problems and challenges for medical staff (ISTH Steering Committee for World Thrombosis Day 2014; Li et al. 2011; Goldhaber and Bounameaux 2012). Due to advancements of medical reform and the gradual formation of the 9073 pension pattern (Jie 2015), the risk of VTE has increased. However, a previous study (Haiyan, Yanru, and Jinping 2020) found that hospital nursing staff members have insufficient knowledge regarding VTE prevention; there are many weak links, and the normalization of preventive nursing behavior is insufficient. Community hospitals lack professional training teachers, and few staff members receive external training. Insufficient training directly leads to a lack of risk awareness, and thus, nurses are unable to actively identify and prevent VTE, thereby leading to considerable safety hazards in clinical work (Yuan et al. 2015; Single 2016; Yanlan et al. 2015). Under the medical consortium model, with tertiary hospitals as the lead unit and the existence of numerous community health service centers, technical assistance (e.g., on‐the‐job training) can be implemented through innovative methods of enhancing nursing resources (Yuli et al. 2021). The Kirkpatrick model is currently the most widely used training evaluation tool and is divided into a reaction layer, a learning layer, a behavior layer and a results layer. This model entails the entire process management and training supervision processes, and it involves comprehensive evaluations (Pengqian and Chong 2022). This study had a one‐group, before‐after, quasi‐experimental design. Relying on the medical consortium, this study applies the Kirkpatrick model to carry out systematic training on VTE prevention among nurses in community hospitals and comprehensively evaluates the effects of the training to provide a reference for future in‐service training programs targeting VTE preventive nursing in community hospitals.

## 2 Objects and Methods

### 2.1 Research Object

The cluster sampling method was used to select 120 clinical nurses who cooperated with a tertiary hospital in Shanghai to build five medical consortium community health service centers. The inclusion criteria were as follows: (1) clinical nurses with a nurse qualification certificate; (2) more than 1 year of ward work experience; (3) voluntary participation in this study. The exclusion criterion was participation in other nursing training‐related research within the past 3 months. The rejection criterion was individuals who could not adhere to the trainers throughout the process as needed. During the training, three subjects were excluded, and 117 subjects were ultimately included in the study, as shown in Table 1.

**TABLE 1:** General characteristics of the study subjects (*n* = 117).

Variable	Number of cases (%)
Age (years)	
20‐29	16 (13.68)
30‐39	75 (64.10)
40‐49	22 (18.80)
50‐59	4 (3.42)
Education	
Technical Secondary School	5 (4.27)
Junior College	37 (31.63)
Undergraduate	75 (64.10)
Title	
Nurse	20 (17.09)
Nurser	57 (48.72)
Supervisory Nurser	38 (32.48)
Deputy Chief Nurser	2 (1.71)
Working duration (years)	
1‐9	27 (23.08)
10‐19	71 (60.68)
20‐29	15 (12.82)
≥30	4 (3.42)

### 2.2 Research Methods

#### 2.2.1 Team Formation

Based on the Delphi expert method, a medical joint prevention VTE care linkage model was developed, and a team of experts (including two chief nurses, two deputy chief nurses, three chief nurses, one chief vascular surgeon, and one doctor‐in‐study) was established by the nursing department of the leading unit of the medical consortium, which was also responsible for the development of the training programs. A thrombosis management specialist was responsible for the implementation of nursing training, assessment, quality supervision, and tracking feedback. A thrombosis management liaison was responsible for recruiting the nurses at community health service centers to participate in the training; communicating with the thrombosis management specialist; and collecting information about training problems, opinions, and feedback from nurses.

#### 2.2.2 Development of a VTE Prevention Training Program for Community Nurses in Medical Consortium

A literature review was conducted, a questionnaire survey was administered, and on‐site supervision of the training needs was performed by the Thrombosis Management Specialist of the main unit of the medical consortium from October to November 2022 at five community health service centers. Based on the discussion and opinions among the expert group, a training program was developed for VTE prevention among community nurses in the medical consortium. The content of the program covered nursing theory skills related to VTE prevention. The duration of the program was 25 h. The main contents are shown in Table 2.

**TABLE 2:** VTE prevention training program for community nurses in the medical consortium.

Module	Training content	Training objectives	Training hours	Training form
Nursing theory related to VTE prevention	Basic overview of venous thromboembolism	Community nurses in the medical consortium understand VTE, improve their awareness of harmfulness, and master knowledge regarding clinical manifestations and emergency treatment methods.	2	Theoretical Teaching
	Risk assessment of venous thromboembolism	Community nurses in the medical consortium master VTE risk assessment methods	2	Theoretical Teaching
	Prevention and treatment of venous thromboembolism	Community nurses in the medical consortium master the common methods and points for attention of basic VTE prevention, physical prevention and drug prevention	2	Theoretical Teaching
	Anticoagulant subcutaneous injection nursing specification	Community nurses in the medical consortium become proficient in anticoagulant subcutaneous injection nursing standards	1	Theoretical Teaching
	New progress in the treatment of venous thromboembolism	Community nurses in the medical consortium understand new advances in VTE treatment	1	Theoretical Teaching
	Interpretation of the latest expert consensus and guidelines for venous thromboembolism	The community nurses in the medical consortium understand the relevant expert consensus on the mechanical prevention of VTE, the prevention and treatment of VTE related to infusion catheters, and the quality evaluation and management guidelines for the prevention and treatment of VTE in hospitals	2	Theoretical Teaching
Nursing skills related to VTE prevention	Ankle pump movement operation	Community nurses in the medical consortium master ankle pump movement methods and operation precautions	1	Operation teaching group practice
	Operation of subcutaneous injection of low molecular weight heparin	Community nurses in the medical consortium master the method of subcutaneous injection of low molecular weight heparin and operation precautions	3.5	Operation teaching group practice
	Operation of putting on and taking off medical pressure socks	Community nurses in the medical consortium master the wearing and tearing‐off methods and operation precautions of medical compression stockings	3.5	Operation teaching group practice
	Operation of air pressure therapeutic instrument	Community nurses in the medical consortium master the method and operation precautions of air pressure therapeutic apparatus	3.5	Operation teaching group practice
	Elastic bandage operation	Community nurses in the medical consortium become proficient in bandaging methods and operation precautions related to elastic bandages	3.5	Operation teaching group practice

*Note*: 1 class hour is 45 min.

#### 2.2.3 Implementation Training

The training teachers include five thrombus management commissioners from the main unit of the medical consortium, all of whom have participated in the VTE nursing training course held by the municipal nursing association and passed the examination administered by the VTE expert team of the hospital. According to the VTE prevention ability training program for community nurses in the medical consortium, the theoretical and practical courses were recorded, and the recordings were sent to the thrombosis liaison officers of each community health service center after being approved by the expert team. The thrombus liaison officer organized and arranged the course training materials (playing and recording courses) once every 2 weeks in the unit for a duration of less than 2 h. The liaison contacted the thrombus management specialist to conduct on‐site checks during the practical courses. The training period lasted from December 2022 to May 2023, and the survey data of the layer and results layer were obtained from June 2023 to November.

#### 2.2.4 Quality Control

The thrombus liaison officer informed the thrombus management specialist about the training schedule every month, the nursing department of each community service center supervised training attendance, and the nursing department of the lead unit organized spot checks on the quality of on‐site training. The thrombosis management specialist strictly reviewed the training attendance. For special cases, the liaison officer issued one‐on‐one training videos for further training. Within 1–3 days after each course, 3–5 questions about course assignments were sent to trainees via the questionnaire to inspect the quality of course training. The VTE preventive nursing WeChat group within the medical consortium was established to promote communication between community health service centers and training teachers, answer questions in a timely manner, and facilitate the sharing of learning materials and work experience.

#### 2.2.5 Evaluation Index

To evaluate the ability of community nurses in the medical consortium to prevent VTE through the four dimensions of the offset model, the response layer was evaluated by a homemade assessment questionnaire that was developed based on two rounds of consultation, discussion, and revision by the nine experts. The content validity of the questionnaire was 0.816. Ten nurses from each of the two community health service centers were randomly selected for the preexperiment, and the Cronbach alpha coefficient was 0.851. The learning layer evaluates theoretical and practical knowledge, and the behavior layer mainly observes the implementation of VTE prevention methods in the daily work of nurses. The results layer involves patient VTE preventive care compliance and standardization as well as feelings reported in the interview.

##### 2.2.5.1 Reaction Layer

According to a review of the literature (Yanqing and Zheng 2014), the questionnaire assesses training objectives, outlines, organization, and management. The questionnaire includes 18 items across five dimensions (Table 3). Each item is scored on a 5‐point Likert scale with the following response options: very satisfied, satisfied, generally satisfied, dissatisfied, and very dissatisfied, corresponding to scores of 5, 4, 3, 2, and 1, respectively. Scores ≥ 3 points indicated overall satisfaction. The maximum scores for the training objectives, outlines, the scores of organization, management and overall situation domains are 10 points, 35 points, 30 points, 15 points, and 10 points, respectively. Additionally, the attendance rate of nurses participating in the training was used as an indicator of the degree of training and learning attitudes.

**TABLE 3:** Questionnaire on satisfaction evaluation of VTE prevention ability training for community nurses in the medical consortium (*n* = 117, number of cases, %).

Dimension	Entries	Very satisfied	Satisfied	Generally satisfied
Training objectives	1. Achieve their desired goals	58 (49.57)	47 (40.17)	12 (10.26)
	2. To achieve the objectives and requirements of the curriculum program	53 (45.30)	53 (45.30)	11 (9.40)
Training outline	1. The content can guide clinical practice and has practical value	65 (55.56)	48 (41.03)	4 (3.41)
	2. The overall curriculum is reasonable	61 (52.14)	50 (42.73)	6 (5.13)
	3. The overall schedule of the course is reasonable	55 (47.0)	49 (41.88)	13 (11.11)
	4. Reasonable arrangement of course hours	59 (50.43)	48 (41.02)	10 (8.55)
	5. The training method is reasonable and conducive to mastering the knowledge	55 (47.0)	49 (41.88)	13 (11.11)
	6. Advanced content, can fill their theoretical gaps	61 (52.14)	53 (45.30)	3 (2.56)
	7. The integrity and continuity of the content system	68 (58.12)	45 (38.46)	4 (3.42)
Training organization	1. Professional level of training teachers	69 (58.97)	43 (36.75)	5 (4.27)
	2. The teaching level of training teachers	69 (58.97)	43 (36.75)	5 (4.27)
	3. Diversity of teacher training methods	57 (48.72)	49 (41.88)	11 (9.40)
	4. Instructor's bedside teaching ability	70 (59.83)	43 (36.75)	4 (3.42)
	5. Teaching equipment meets the demand	10 (8.55)	58 (49.57)	49 (41.88)
	6. Teaching venues meet the demand	10 (8.55)	49 (41.88)	58 (49.57)
Training management	1. Training management ability	62 (52.99)	47 (40.17)	8 (6.84)
	2. Training management level of problem solving	65 (55.56)	43 (36.75)	9 (7.69)
	3. Professional training management	63 (53.85)	46 (39.32)	8 (6.83)
General situation	1. Overall satisfaction with training	71 (60.68)	41 (35.04)	5 (4.27)
	2. Meet the needs of clinical work	75 (64.10)	36 (30.77)	6 (5.13)

##### 2.2.5.2 Learning Layer

The theoretical knowledge assessment was unified by the training teachers according to the VTE prevention ability training program for community nurses in the medical consortium and was reviewed by an expert team. The assessment included basic VTE knowledge, basic prevention knowledge, physical prevention, and drug prevention‐related knowledge. The total score on the assessment was 100 points. The skill operation assessment was based on the unified operation scoring standard and was formulated by the nursing department of the leading unit. Two thrombosis management specialists conducted the assessment and scoring at the same time. The total score of the five skills in the assessment plan was 100 points, and the final score was obtained by taking the average score of the five operations.

##### 2.2.5.3 Behavioral Layer

The hospitalized adult patients whose VTE prevention and nursing group standards were issued by Chinese nurses were evaluated. We designed an implementation questionnaire to assess VTE nursing prevention for patients by nurses, with 10 items in total. Each item was scored on a 5‐point scale, thus yielding a maximum score of 50 points (Table 4). Three months after the training, the nursing department of the lead unit cooperated with the thrombosis prevention nursing management specialist to evaluate the daily VTE preventive nursing behaviors of 117 nurses via random on‐site quality control. Additionally, an on‐site investigation was carried out on the VTE risk assessment rate and the implementation rate of VTE preventive measures for the patients in charge of the trained nurses within 24 h.

**TABLE 4:** Questionnaire on the implementation of VTE prevention for patients by nurses.

Serial number	Project content	Score
1	If there is no taboo, the lower limbs of bedridden patients should be raised so that the lower limbs are 20–30 cm higher than the heart plane, and hard pillows and excessive hip flexion should be avoided under the knee	5
2	Bedridden patients should be instructed and assisted in active and passive movements of the lower extremities, including ankle pump exercises and quadriceps functional exercises	5
3	Ankle pump exercises to guide the correct movement	5
4	Patients should be guided to get out of bed as soon as possible according to the recovery of the disease	5
5	On the premise of meeting the treatment needs, we should try to choose the infusion device with the smallest diameter and the least trauma	5
6	The placement and maintenance of all kinds of intravenous catheters should be standardized, and venous puncture of lower limbs and affected limbs should be avoided as far as possible	5
7	If the condition permits, the patient should be instructed to drink 1500–2500 mL of water every day	5
8	Patients should be instructed to quit smoking and limit alcohol, balanced diet, weight control, blood sugar, blood lipids, and not sedentary	5
9	Implement the risk assessment of thrombosis in patients and conduct patrol in each class	5
10	Strengthen communication with doctors and implement physical prevention and drug prevention measures when necessary	5

##### 2.2.5.4 Results Layer

The compliance with and standardization of VTE preventive care for inpatients in community health service centers within the medical consortium was evaluated. The compliance and standardization of VTE preventive nursing skills among patients were evaluated by the management specialist of thrombosis prevention and nursing, respectively, via on‐site quality control at 1 month and 3 months after the training. According to the nursing quality control standard of thrombosis prevention in the main unit, patients were randomly selected during quality control (excluding those who cannot carry out autonomous activities or follow the doctor's instructions). If patients fail to carry out VTE preventive nursing measures (i.e., noncompliance) or if there is compliance with VTE preventive nursing measures but there is one measure that is not implemented according to the standard, then the preventive skills are considered to be not standard. Finally, the compliance rate and standard rate were calculated. Three months after the training, a semistructured interview was conducted with 15 randomly selected nurses via phenomenological research. The interviews mainly focused on the effect of the VTE prevention ability training of community nurses in this medical consortium. The interviews lasted for 15–20 min and assessed topics such as whether the training improved the individual's working ability, what specific benefits the training confers, what shortcomings there are in community VTE care, and what help is needed.

### 2.3 Statistical Methods

SPSS23.0 software was used for statistical analysis, and all the data analyses were crosschecked by two people. The quantitative data were compared with paired *t* tests, and the categorical data were compared with the χ^2^ test. *p* < 0.05 indicated a statistically significant difference.

## 3 Results

### 3.1 Evaluation Results of the Response Layer With Respect to VTE Prevention Ability Training for Community Nurses in the Medical Consortium

The attendance rates for theoretical training and skills training were 98% and 100%, respectively. The degree of satisfaction of the trainees was relatively high. A total of 117 copies of the “evaluation questionnaire on the satisfaction of VTE prevention ability training for community nurses in the medical union” were distributed, and 117 valid responses were obtained. There were no dissatisfied or very dissatisfied respondents (Table 3 for details).

### 3.2 Evaluation Results of the Learning Layer With Respect to VTE Prevention Ability Training for Community Nurses in the Medical Consortium

The theoretical knowledge before and after the training improved from 44.34 ± 13.621 to 75.15 ± 10.914, and the operational skills improved from 58.509 ± 5.8832 to 78.269 ± 6.3224. These differences were statistically significant (*p* = 0.001), as shown in Table 5.

**TABLE 5:** Comparison of learning level results of VTE prevention ability training for community nurses in the medical consortium (*n* = 117 $\bar{x\;}\pm \;s$, points).

Time	Theoretical knowledge	Skill operation
Before training	44.34 ± 13.621	58.509 ± 5.8832
After training	75.15 ± 10.914	78.269 ± 6.3224
T value	−65.688	−74.269
*p* value	<0.001	<0.001

### 3.3 Evaluation Results of the Behavior Layer With Respect to VTE Prevention Ability Training for Community Nurses in the Medical Consortium

Before training and 3 months after training, the daily VTE preventive nursing behaviors of 117 nurses were investigated, and the VTE preventive nursing execution survey score of patients increased from 19.231 ± 4.1323 to 44.615 ± 3.2927. This difference was statistically significant (*p* < 0.001), as shown in Table 6. The VTE risk assessment rate and the implementation rate of VTE preventive measures increased from 12% and 25%, respectively, before training to 95% and 97%, respectively, after training.

**TABLE 6:** Comparison of behavioral level results of VTE prevention ability training for community nurses in the medical consortium (*n* = 117 $\bar{x}\;\pm \;s$, points).

Time	Score
Before training	19.231 ± 4.1323
After training	44.615 ± 3.2927
*T* value	−199.293
*p* value	<0.001

### 3.4 Evaluation Results of the Results Layer With Respect to VTE Prevention Ability Training for Community Nurses in the Medical Consortium

After 3 Months of Training, the patient's ankle pump movement compliance rate and standard rate increased from 14.61% and 0%, respectively, before training to 85.26% and 60%, respectively, after training. These differences were statistically significant (*p* < 0.001), as shown in Table 7. According to the collation and analysis of the semistructured interview data, the trained nurses believe that the VTE prevention training increased risk awareness, improved preventive nursing ability, effectively improved patient safety, and improved nurses’ sense of value, hope for long‐term help and guidance.

**TABLE 7:** Comparison of compliance with and standardization of ankle pump exercise among patients.

	Pretraining (*n* = 89)	1 month after training (*n* = 83)	3 months after training (*n* = 95)	*χ* ^2^	*p*
Rate of compliance (%)	14.61 (13)	74.7 (62)	85.26 (81)	107.573^a^	<0.001
Standard rate (%)	0 (0)	43.37 (36)	60 (57)	23.411^a^	<0.001

^a^ Representing that the expected count of 0 cells (0.0%) is less than 5.

## 4 Discussion

### 4.1 The Kirkpatrick Model Is Feasible and Effective for Improving the Effect of VTE Training on Nurses in the Medical Consortium

The ultimate goal of training is to apply the knowledge learned to practice and better carry out the work. The Kirkpatrick model can manage, supervise and evaluate the whole process of training in a comprehensive manner, including the reaction layer, the learning layer, the behavior layer, and the results layer, thereby improving the quality of the training process and the quality of the results.

This model not only focuses on the satisfaction of the trainees but also pays attention to the application of knowledge and the changes in attitudes and behavior (Wei 2018). The application of the Kirkpatrick model to VTE prevention training among nurses in the medical consortium can effectively improve their theory and skills, thereby improving their self‐prevention compliance and standardization.

### 4.2 Reaction Layer Evaluation

The response layer measures the willingness to learn and the preference for the training course by the satisfaction and attendance of the participants. The results of this study show that the attendance rate of participating nurses was ≥98%, indicating that nurses were highly interested in VTE preventive nursing courses and were very willing to take the initiative to learn; of course, this is also related to the lack of VTE‐related knowledge reserves among nurses in the early stage of their career and the lack of regular training. The response level survey showed that the trainees were very satisfied with the professional level and teaching level of the training teachers, the number of satisfied students was as high as 95.73%, and the bedside teaching ability of the teachers reached 96.58%. This should be the main reason for enhancing the interest of the trainees in learning, and it is also the key factor in achieving good results in later training. The teachers of this training are all thrombus management specialists of the main unit of the medical consortium. There is a solid level of professional knowledge and rich experience in nursing related to VTE, which suggests that the establishment of a professional training team is also a key factor in improving the effect of VTE prevention training. However, in the survey, 41.88% and 49.57% of people were satisfied with the “teaching equipment” and “teaching site,” respectively. Obviously, the construction of teaching equipment and sites in community hospitals of medical consortiums is uneven, and this paper puts forward some thoughts on whether the homogenization of teaching hardware equipment in medical consortiums should be carried out.

### 4.3 Learning Level Evaluation

The learning layer is the dimension related to the knowledge change of the trainee before and after the training. The nurses’ understanding and mastery of the relevant training content can be determined through the assessment of theory and skills, respectively. In this study, the nurses' VTE preventive nursing theoretical knowledge and operational skills assessment results improved after training, indicating that the translated their knowledge into practice and obtained a solid foundation for improving the level of VTE prevention and care for patients. The increase in theoretical knowledge improvement was greater than the improvement in practical knowledge, which suggests that further research can be conducted on the knowledge system composition of on‐the‐job nurses, especially on‐the‐job nurses in the community, to provide a theoretical basis for better implementation of job competency‐oriented on‐the‐job training.

### 4.4 Behavior Layer Evaluation

The behavior level mainly inspects the behavior changes of the trainees at work after the training to judge the influence of the knowledge learned on the actual work. The evaluation time should be at least 3 months after the training. (Wei 2018). In this study, after 3months of training, the nurses' VTE nursing prevention implementation survey score improved, and the VTE risk assessment rate and the implementation rate of VTE preventive measures also increased from 12% and 25%, respectively, before training to 95% and 97%, respectively, after training. It is suggested that nurses can apply the knowledge learned in practical work after this training, effectively improve their clinical practice ability, and indirectly prove the effectiveness of this training program.

### 4.5 Results Layer Evaluation

The results layer is mainly used to evaluate the improvement of training on the quality of work from the organizational point of view but also to judge the changes that training may bring to hospitals, patients, and nurses (Huiling, Bin, and Yan 2021). This study revealed that after 3 months of training, the compliance rate and standard rate of ankle pump movement in patients increased from 14.61% and 0%, respectively, before training to 85.26% and 60%, respectively, after training, thus effectively enhancing patients’ ability to prevent VTE through action consciousness and standardization to improve patient safety and indirectly improve the quality of hospital medical safety. The nurses also realized that after training, the awareness of VTE risk increased, and preventive nursing improved, which not only ensured the safety of patients but also ensured the nurses’ occupational safety. It is suggested that the homogenization of nursing training in the medical consortium system should be carried out in a sustainable way.

## 5 Summary

This study applies the offset model to the VTE theoretical knowledge and skills training of community nurses in the medical consortium; implements homogenized nursing prevention standard training; and refines, quantifies, and concretes the evaluation system from four dimensions, thereby making the evaluation of the training effect scientific, measurable and applicable. In the future, based on the VTE nursing linkage mode of the medical consortium, we can implement VTE nursing training, assessment, and quality supervision for community hospital nurses in a standardized manner to continuously improve the standard prevention rate of VTE and reduce the incidence of VTE in patients, thereby ensuring patient safety.

## Ethics Statement

First, this research does not involve the collection or analysis of human or animal experiment data. Second, this research was reviewed and qualified by the Ethics Committee of Zhoupu Hospital in Pudong District, Shanghai, and the approval number is ZPYYLL‐2018‐02.

## Conflicts of Interest

The authors declare that they have no competing interests.

## Data Availability

Data sharing is not applicable to this article as no new data were created or analyzed in this study.
